# Genes related to N6-methyladenosine in the diagnosis and prognosis of idiopathic pulmonary fibrosis

**DOI:** 10.3389/fgene.2022.1102422

**Published:** 2023-01-04

**Authors:** Jingcheng Zhang, Ying Zhang, Ziyuan Wang, Jiachao Zhao, Zhenyu Li, Keju Wang, Lin Tian, Baojin Yao, Qibiao Wu, Tan Wang, Jing Wang

**Affiliations:** ^1^ Northeast Asia Research Institute of Traditional Chinese Medicine, Changchun University of Chinese Medicine, Changchun, China; ^2^ Department of Respiratory, The Affiliated Hospital to Changchun University of Chinese Medicine, Changchun, China; ^3^ College of Traditional Chinese Medicine, Changchun University of Chinese Medicine, Changchun, China; ^4^ College of Integrated Traditional Chinese and Western Medicine, Changchun University of Chinese Medicine, Changchun, China; ^5^ State Key Laboratory of Quality Research in Chinese Medicines, Faculty of Chinese Medicine, Macau University of Science and Technology, Macau, China; ^6^ Guangdong-Hong Kong-Macao Joint Laboratory for Contaminants Exposure and Health, Guangdong University of Technology, Guangzhou, China; ^7^ Zhuhai MUST Science and Technology Research Institute, Zhuhai, China

**Keywords:** idiopathic pulmonary fibrosis, N6-methyladenosine, *METTL14*, diagnosis, prognosis

## Abstract

**Introduction:** Idiopathic pulmonary fibrosis (IPF) is a chronic progressive pulmonary fibrotic disease with unknown etiology and poor outcomes. It severely affects the quality of life. In this study, we comprehensively analyzed the expression of N6-methyladenosine (m6A) RNA methylation regulators using gene expression data from various tissue sources in IPF patients and healthy volunteers.

**Methods:** The gene expression matrix and clinical characteristics of IPF patients were retrieved from the Gene Expression Omnibus database. A random forest model was used to construct diagnosis signature m6A regulators. Regression analysis and correlation analysis were used to identify prognosis m6A regulators. Consensus cluster analysis was used to construct different m6A prognosis risk groups, then functional enrichment, immune infiltration and drug sensitivity analysis were performed.

**Result:** Five candidate m6A genes from lung tissue were used to predict the incidence, and the incidence was validated using datasets from bronchoalveolar lavage fluid (BALF) and peripheral blood mononuclear cells. Subsequently, the BALF dataset containing outcomes data was used for the prognosis analysis of m6A regulators. *METTL14*, *G3BP2*, and *ZC3H13* were independent protective factors. Using correlation analysis with lung function in the lung tissue-derived dataset, *METTL14* was a protective factor in IPF. Based on *METTL14* and *G3BP2*, a consensus cluster analysis was applied to distinguish the prognostic m6A regulation patterns. The low-risk group’s prognosis was significantly better than the high-risk group. Biological processes regulated by various risk groups included fibrogenesis and cell adhesion. Analysis of immune cell infiltration showed upregulation of neutrophils in the m6A high-risk group. Subsequently, five m6A high-risk group sensitive drugs and one m6A low-risk group sensitive drug were identified.

**Discussion:** These findings suggest that m6A regulators are involved in the diagnosis and prognosis of IPF, and m6A patterns are a method to identify IPF outcomes.

## 1 Introduction

Idiopathic pulmonary fibrosis (IPF) is a chronic, progressive fibrotic lung disease with unknown causes. The incidence of IPF is rising, and the prognosis are extremely poor, severely affecting the quality of life (Mei et al., 2021). Cardinal symptoms of IPF include progressive dyspnea and dry cough (Hochhegger et al., 2019; Janowiak et al., 2022). Currently, there is no effective treatment for IPF, and the median survival time is 3 to 5 years. The overall survival is poor, and there are individual differences. Despite significant progress in understanding its pathogenesis, it remains difficult reliably to predict the disease course and individual patient responses to treatment. Serum biomarkers such as osteonectin, matrix metallopeptidase-7, intercellular adhesion molecule-1, and periostin are somewhat predictive for diagnosis, prognosis, and treatment responses (Drakopanagiotakis et al., 2018; Clynick et al., 2022). Nevertheless, there remains a need for biomarkers that predict IPF onset and prognosis.

N6-methyladenosine (m6A) RNA modification involves methyltransferase catalyzing the methylation of adenine at the 6N position. The methyltransferase complex catalyzes the formation of m6A (Bokar et al., 1994), including three modifications. The m6A readers include *EIF3A*, *FMR1*, *G3BP1*/*2*, *HNRNPA2B1*, *HNRNPC*, *HNRNPG*, *LRPPRC*, *IGF2BP1/2/3*, *PRRC2A*, *RBMX*, *YTHDC1*/*2*, *YTHDF1/2/3*, and *SND1* (Lan et al., 2019; Lan et al., 2021; Zhang et al., 2021; Li et al., 2022); The m6A writers include *CBLL1*, *KIAA1429*, *METTL3*, *METTL5*, *METTL14*, *METTL16*, *PCIF1*, *RBM15*, *RBM15B*, *VIRMA*, *WTAP*, *ZC3H13*, *ZCCHC4*, and *HAKAI* (Wang et al., 2015; Xiao et al., 2016; Wu et al., 2018; Lan et al., 2019; Lan et al., 2021; Zhang et al., 2021; Li et al., 2022; Qu et al., 2022); m6A erasers include *FTO*, *ALKBH3* and *ALKBH5* (Lan et al., 2019; Gao et al., 2021; Lan et al., 2021; Li et al., 2022). Writers and erasers are in the nucleus, where m6A binds a specific nuclear reader, affecting mRNA shearing and other nuclear processes. After export to the cytoplasm, m6A binds to a specific cytoplasmic reader, affecting mRNA stability, translation, and localization (Zaccara et al., 2019; Jiang et al., 2021).

Regulators of m6A RNA methylation modify the course of lung cancer and pneumonia. The m6A regulators that aid the diagnosis and prognosis of IPF haves not yet been discovered. Therefore, we performed a preliminary analysis of m6A-regulated genes based on high-throughput sequencing data of clinical samples. RNA methylation is the most abundant modification in eukaryotes; m6A regulators may be involved in IPF pathogenesis and progression and influence outcomes by regulating biological processes and immune infiltration. In this study, we identified the m6A regulators for IPF diagnosis and outcomes and explored the biological processes regulated by m6A regulators.

## 2 Materials and methods

### 2.1 Collection of clinical information and data sets

The gene expression matrix and clinical characteristics of IPF patients were retrieved from the Gene Expression Omnibus (GEO) database (https://www.ncbi.nlm.nih.gov/geo/), and a total of 505 IPF patients and 147 healthy volunteers were included. The bronchoalveolar lavage fluid (BALF) came from the GSE70866 series, including IPF patients (*n* = 176) and healthy volunteers (*n* = 20), which were detected using the GPL14550 and GPL17077 platforms, respectively (Prasse et al., 2019). The lung tissue came from the GSE47460 series, including interstitial lung disease patients (*n* = 254) and healthy volunteers (*n* = 108), which were tested using the GPL6480 and GPL14550 platforms, respectively (Kim et al., 2015; Peng et al., 2016; Anathy et al., 2018). The source of peripheral blood mononuclear cells (PBMCs) was the GSE28221 series, including IPF patients (*n* = 75) and healthy volunteers (*n* = 19), which were detected using the GPL6480 platform (Herazo-Maya et al., 2013; Huang et al., 2015). Due to the different detecting platforms of samples, the batch effect detected by the platforms was eliminated using the ComBat method in the “SVA” R package for data from the same histologic origin. Principal component analysis (PCA) was used to determine the goodness of batch correction.

### 2.2 m6A identification

Based on previous studies, 36 m6A regulators were considered, including 19 readers, 14 writers, and three erasers. The m6A regulators described above were extracted using the “limma” package in R software. The Wilcoxon test was used to determine whether there were significant changes between IPF patients and healthy volunteers. The “ggpubr” package was employed to draw expression boxplots, and the “pheatmap” package was used to draw a visual m6A expression heatmap.

### 2.3 Random forest (RF) and support vector machine (SVM) model testing and development of nomogram models

We constructed machine learning classifiers of RF and SVM to predict the reliability of IPF occurrence (Noble, 2006; Altmann et al., 2010). A hyperplane was identified to distinguish IPF patients from healthy volunteers. Then, we established a nomogram to predict the occurrence of IPF in different GEO series based on five selected m6A regulators. Statistical analysis was performed using the R packages “rms” and “rmda.” To assess model accuracy and validity, plot calibration curves, decision curve analysis (DCA), and receiver operating characteristic (ROC) curves. Finally, the nomogram formula of the GSE47460 series was brought into GSE70866 and GSE28221 series for verification. ROC curves were drawn to calculate the area under the curve (AUC) and 95% confidence interval (CI).

### 2.4 Cox proportional hazards regression model, least absolute shrinkage and selection operator (LASSO) regression model

The 31 m6A-related genes of the BALF-derived GSE70866 series were analyzed using univariate Cox regression analysis. Multi-variables faced the risk of over-fitting. LASSO regression was applied to analyze the influence of the survival status of these three variables, and the parameter λ was used to adjust the model complexity to obtain a less variable and more representative combination. Prognostic high-risk and low-risk clusters were obtained according to the risk score using LASSO regression. The 33 m6A-related genes of the PBMC-derived GSE28221 series were analyzed using univariate Cox regression analysis.

The risk score of IPF patients can be used as an independent prognostic factor. Among the clinical data, age, gender, and global alignment and proportion (GAP) score were included as independent variables in univariate and multivariate Cox regression analyses to clarify independent prognostic factors for IPF patients. To balance the statistical data, we used 1000× risk scores. To perform these analyses, we used the “forestplot,” “survival,” and “glmnet” packages.

### 2.5 Spearman correlation analysis

Spearman correlation analysis was performed on the lung function using the diffusing capacity of the lungs for carbon monoxide (DLCO) and m6A regulators (|R| > 0.2, *p* < 0.001) in the GSE47460 series using the “limma” package. Spearman correlation analysis was performed on *METTL14*, *G3BP2*, and *ZC3H13* with other m6A regulators (|R| > 0.4, *p* < 0.001) in the GSE70866 series. The “ggplot2,” “ggpubr,” and “ggExtra” packages were used for visual scatter plot drawing.

### 2.6 Consensus cluster analysis, PCA and survival analysis

To determine whether the outcomes-related m6A regulators *METTL14*, *G3BP2*, and *ZC3H13* are associated with IPF, series from GSE70866 were divided into groups according to the consensus levels of m6A regulators using the “ConsensusClusterPlus” package. Outputs included consensus cumulative distribution function (CDF) plots, the relative change in area under the CDF curve, and the consensus matrix. PCA was used to determine the fitness of classification. Kaplan-Meier survival analysis was used to evaluate the difference in overall survival between different m6A types using the “survival” package.

### 2.7 Differentially expressed genes (DEGs) analysis and Gene Ontology (GO) functional annotation and kyoto encyclopedia of genes and genomes (KEGG) pathway analysis of m6A regulators

The “limma” package and the “VennDiagram” package were used to identify DEGs associated with different m6A modifications. Adjusted *p*-values <0.05 and |log2FC| > 1 were considered as DEGs, and volcano plots were drawn for all genes. The “clusterProfiler” package was used to analyze GO functional annotation, including the biological process (BP), celluar component (CC), molecular function (MF) terms, and KEGG pathway analysis. The significance criterion was set at *p*-value <0.05. The results were plotted using a bioinformatics tool (https://www.bioinformatics.com.cn) based on the R language (version 4.03) (Ashburner et al., 2000; Kanehisa and Goto, 2000; Gene Ontology, 2021).

### 2.8 Immune infiltration assay

Initially, we used single-sample gene set enrichment analysis (ssGSEA) to quantify the relative abundance of 23 immune cell types in the immune microenvironment across different m6A clusters. Panels of specific functional genes are used to mark each immune cell type. The relative abundance of each immune cell type was calculated from the enrichment fraction of gene set expression in transcriptome sequencing in ssGSEA analysis and normalized to a uniform distribution. Next, based on the results of the immune-related analysis, a boxplot of differentially expressed immune cells of different m6A clusters was drawn, and an association heatmap was drawn by using m6A regulators and immune cell expression abundance for correlation analysis.

### 2.9 Potential target drug analysis of different m6A clusters

The “pRRophetic” package was used to predict potential drugs sensitive to IPF patients with different m6A risk groups. Drug susceptibility was assessed by the half-maximal inhibitory concentration.

## 3 Results

### 3.1 Identification of signature m6A regulators genes in the diagnosis of IPF

**FIGURE 1:**
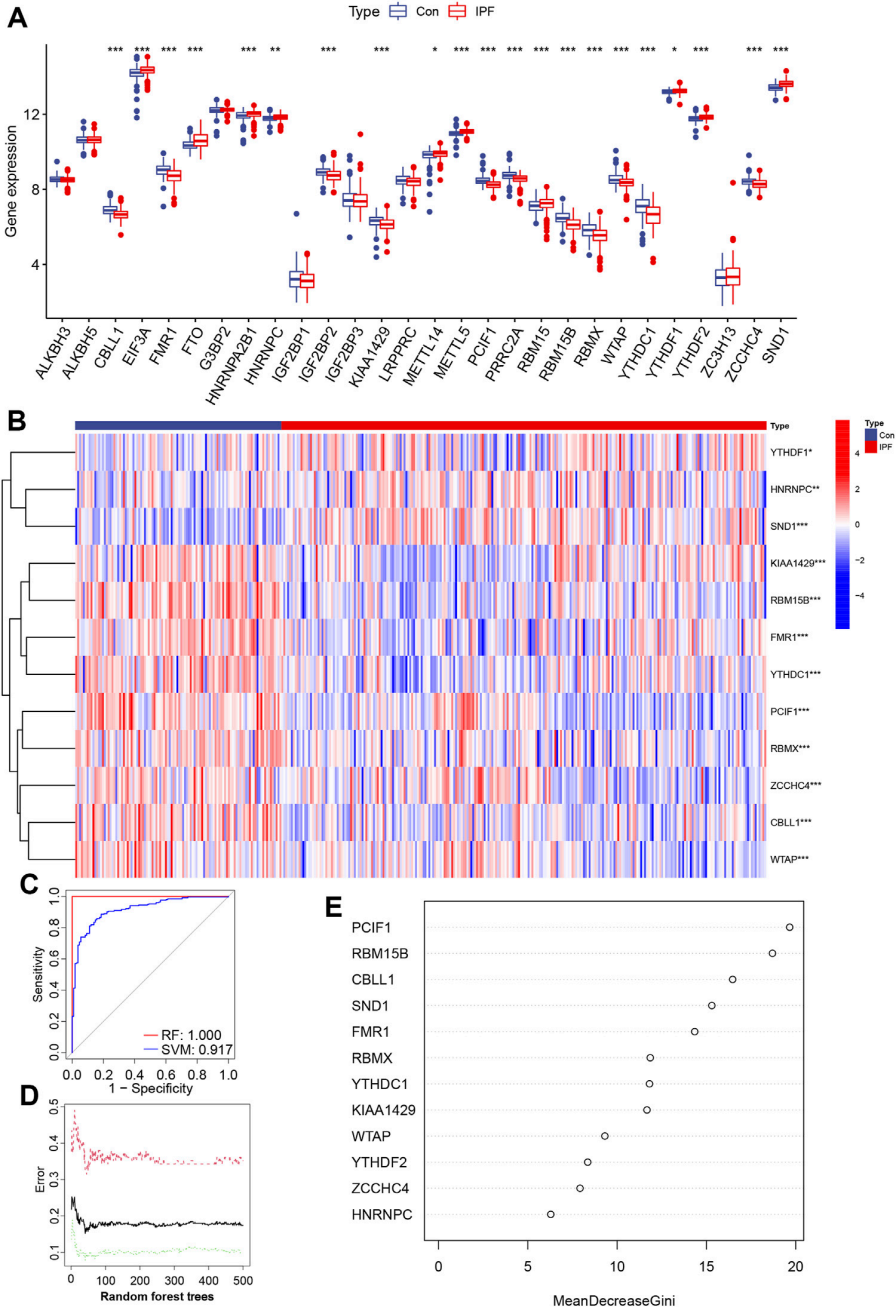
GSE47460 series profile and RF and SVM model construction of m6A regulators in IPF and healthy volunteers (Con) **(A)** Histogram of 28 m6A regulators between IPF and Con. **(B)** Heatmap of differential expression of 12 m6A regulators. **(C)** AUC of the RF and SVM model, SVM is 0.917, RF is 1.000. **(D)** Correlation between the error and number of trees of Con, IPF, and both. **(E)** The importance scores of 12 m6A modulators were calculated based on the RF model. (*p < 0.05, **p < 0.01, ***p < 0.001).

To investigate the differentially expressed m6A gene between IPF patients and healthy people, according to the detection ratio of the groups, we selected the GSE47460 series derived from lung tissue for differentially expressed m6A gene analysis. After batch correction, the two data sets with the same sample source were merged, and the m6A genes were extracted (Supplementary Figures S1A–H). Due to the small number of healthy volunteers, only differentially expressed m6A genes with opposite expression trends in the GSE70866 and GSE47460 series were excluded. We extracted 21 differentially expressed m6A genes from the GSE47460 series, eight DEGs with the opposite expression trend, and one undetected m6A gene of GSE47460 was removed (Figure 1A). We obtained 12 reliable and significantly differentially expressed m6A regulators, including six writers and six readers, and presented the heatmap of differentially expressed m6A regulators (Figure 1B).

Modeling of IPF disease-associated m6A regulators was performed using differentially expressed m6A regulators. Simultaneous RF and SVM models were established in the GSE47460 series to identify m6A regulators characterized by IPF. “ROC curve,” “residual box plot,” and “residual reverse cumulative distribution” suggested that the RF model was more suitable than the SVM model for constructing m6A genes related to pathogenesis (Figure 1C and Supplementary Figures S1I,J). The point with the smallest cross-validation error constructed by RF trees was used as the best model, and the importance scores of 12 m6A regulators were calculated (Figure 1D). The top five m6A regulators (*PCIF1*, *RBM15B*, *CBLL1*, *SND1*, and *FMR1*) were used to build a nomogram to predict prevalence in healthy volunteers and IPF patients (Figure 1E).

**FIGURE 2:**
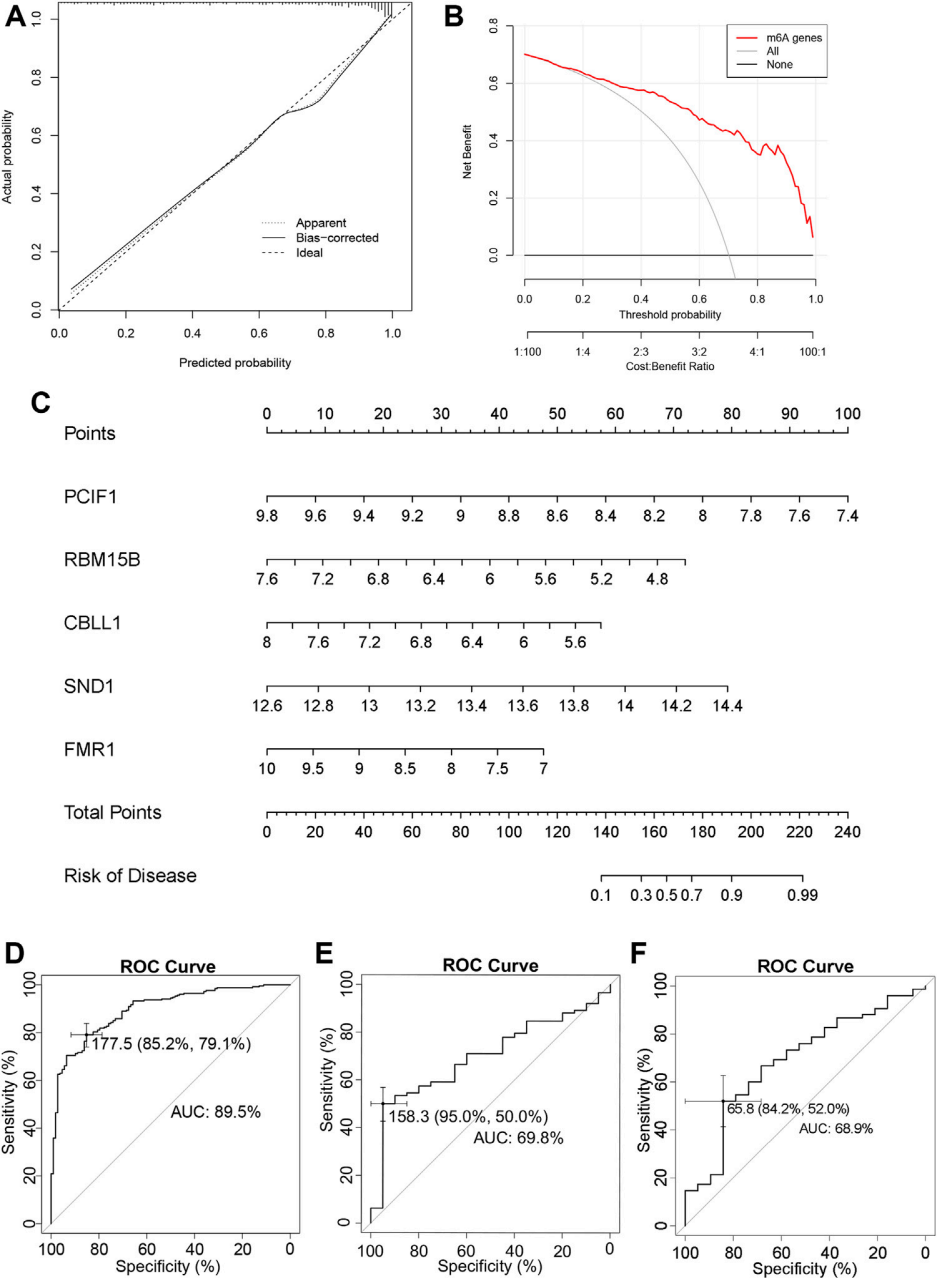
Nomogram to build the prevalence model of IPF **(A)** The calibration curve of the nomogram. **(B)** DCA of the nomogram. **(C)** Nomogram of predicted prevalence according to gene score of GSE47460 series. **(D)** Nomogram predicting the ROC of prevalence in the GSE47460 series. **(E)** Nomogram predicting the ROC of prevalence in the GSE70866 series. **(F)** Nomogram predicting the ROC of prevalence in the GSE28221 series.

The calibration curve can be seen that the “apparent” dotted line corresponding to the entire cohort and the bootstrap corrected solid line are close to the “Ideal” dotted line, indicating that the model has predictive performance (Figure 2A). The DCA method was used to draw a “decision curve” to evaluate the relationship between the nomogram and the gene score to predict the benefits and risks of various cut points in the IPF prevalence model. Figure 2B shows that the selected m6A genes have better benefits at various threshold probabilities.

An m6A gene-associated nomogram of the GSE47460 derived from lung tissue series was then drawn (Figure 2C). The ROC curve indicated the sensitivity and specificity of the diagnosis. The AUC of the SE47460 series was 89.5% (95% CI: 86.19%–92.89%), suggesting a high prediction accuracy (Figure 2D). Then we brought the five m6A regulators into the GSE70866 and GSE28221 to establish the diagnosis-related nomogram construction. The AUC value of the ROC curve was 78.5% (Supplementary Figures S1K,L) and 80.8% (Supplementary Figures S1M,N).

To validate the nomogram constructed based on the lung tissue-derived GSE47460 series (Figure 2C), we used 196 patients from the BALF-derived GSE70866 series and 94 patients from the PBMC-derived GSE28221 series to perform IPF incidence prediction validation and draw ROC curves. The AUCs for the GSE70866 and GSE28221 series were 69.8% and 68.9%, respectively (Figures 2E,F). The source of BALF is lung tissue, and the predictive accuracy was better. There were few samples in the GSE70866 and GSE28221 series; a larger sample size is needed. These findings suggest that *PCIF1*, *RBM15B*, *CBLL1*, *SND1*, and *FMR1* can be used as the characteristic m6A regulators of IPF, and they have diagnostic significance in lung tissue and PBMC.

### 3.2 Identification of signature genes for m6A regulators on outcomes in IPF

**FIGURE 3:**
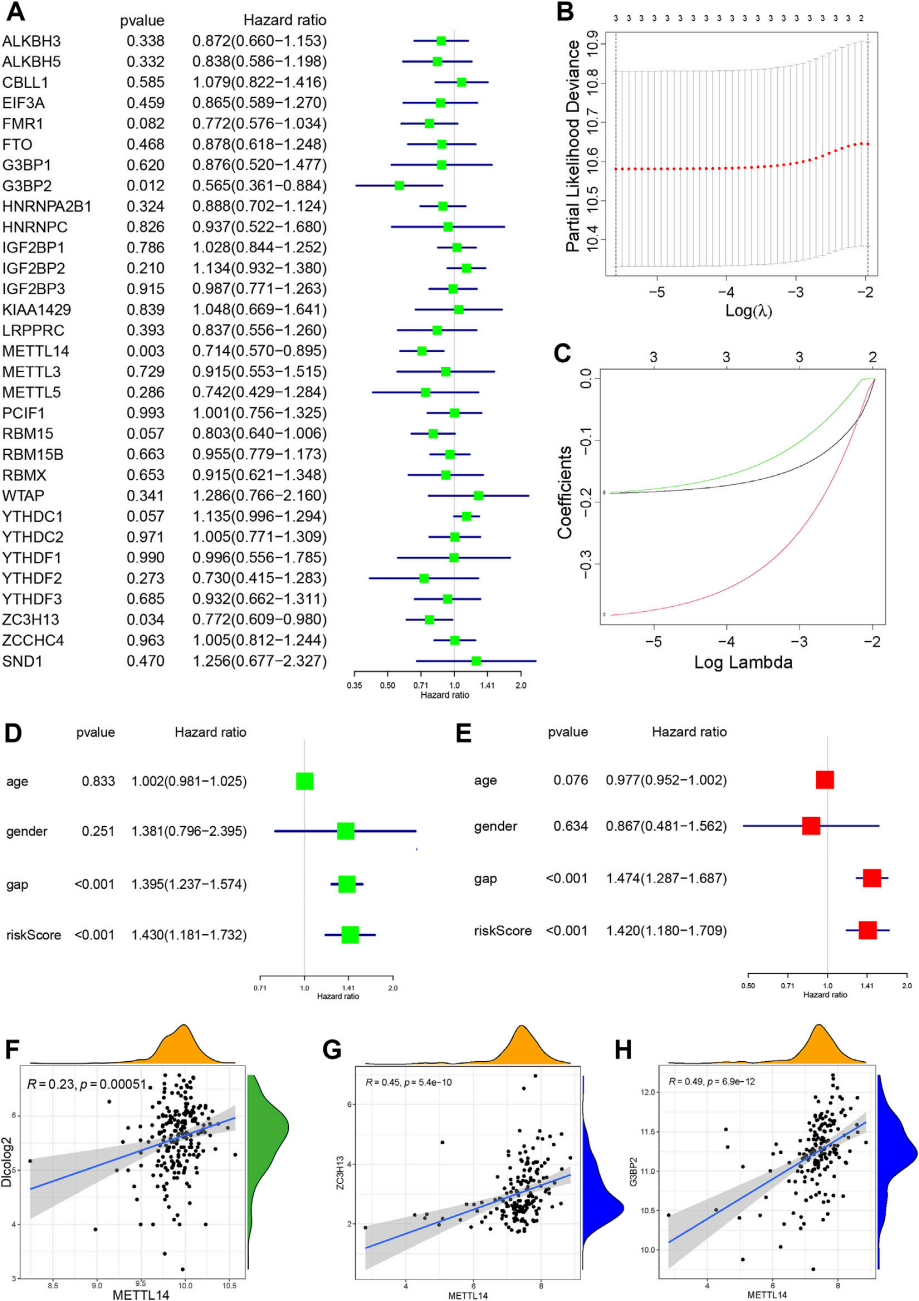
Identification of characteristic genes related to IPF outcomes **(A)** Forest plot of three m6A regulators with *p* < 0.05 by univariate Cox regression. **(B)** Selection of the optimal parameter (lambda) in the LASSO regression model. **(C)** LASSO coefficient profiles of the three m6A regulators in the GSE70866 series. **(D)** Forest plot of m6A risk score and three clinical factors by univariate Cox regression. **(E)** Forest plot of m6A risk score and three clinical factors by multivariate Cox regression. **(F)** Correlation between *METTL14* and DLCO (|R| > 0.2 and *p* < 0.001). **(G)** Correlation analysis of *METTL14* with *ZC3H13*. **(H)** Correlation analysis of *METTL14* with *G3BP2* (|R| > 0.4 and *p* < 0.001).

To clarify the impact of m6A-related genes on the outcomes of IPF, the clinical information provided by the BALF-derived GSE70866 series was used to perform univariate Cox regression analysis on the 31 m6A regulators extracted from the data set (Figure 3A). Three protective m6A regulators (*p* < 0.05) (*METTL14*, *G3BP2*, and *ZC3H13*) were identified. LASSO regression was employed to identify these regulators, and risk scores were calculated (Figures 3B,C). We used univariate Cox regression models to compare the effects of age, gender, GAP, and m6A regulator risk score on patient survival. Figure 3D shows that GAP (HR 1.395, 95% CI: 1.237–1.574, *p* < 0.001) and the m6A regulator risk score (HR 1.430, 95% CI: 1.181–1.732, *p* < 0.001) predict IPF prognosis.

Multivariate Cox regression analysis was performed to control for the influence of confounding factors and to analyze the influencing factors with independent effects. Figure 3E shows that the GAP score (HR 1.474, 95% CI: 1.287–1.687, *p* < 0.001) and m6A regulator risk score (HR 1.420, 95% CI: 1.180–1.709, *p* < 0.001) remained IPF predictors. The BALF-derived GSE28221 series was used to perform univariate Cox regression analysis on the 33 m6A regulators extracted from the data set (Supplementary Figures S2A). *YTHDC1* was a protective m6A regulator (*p* = 0.048). Considering the different sources of PBMC and BALF, PBMC may be affected by several organs and tissues through blood circulation, and the sample size of GSE28221 is small. The evidence for identifying IPF outcomes related m6A regulators in PBMC is insufficient, and more clinical data support is needed.

To identify the optimal IPF prognosis-related m6A modulators, we used lung function data from the GSE47460 series. Lower DLCO indicates worse lung function and is associated with poor outcomes. Spearman correlation analysis was used to analyze the correlation between DLCO and m6A regulators (|R| > 0.2, *p* < 0.01), and we found six genes in the GSE47460 series that were weakly correlated with DLCO. *KIAA1429*, *FMR1*, *PCIF1*, *YTHDC1*, and *METTL14* were positively correlated with DLCO and considered potential protective factors for prognosis. *YTHCF1* negatively correlated with DLCO and was a potential risk factor for poor prognosis (Supplementary Figures S3A–E). The expression of *METTL14* was positively correlated with DLCO (R = 0.23) (Figure 3F) and was identified as a critical variable for outcomes in the GSE70866 and GSE47460 series. The expression of *YTHDC1* was positively correlated with DLCO, consistent with the PMBC-derived GSE28221 series.

We determined whether *METTL14, G3BP2*, and *ZC3H13* levels correlate with other m6A genes. *METTL14*, *G3BP2*, and *ZC3H13* are protective factors for prognosis, and *METTL14* is positive correlations with *ZC3H13* and *G3BP2* (Figures 3G,H). Besides, five m6A genes were moderately correlated with the *METTL14* expression level, including three readers (*IGF2BP3*, *LRPPRC*, and *YTHDC2*) and the two writers (*KIAA1429* and *WTAP*) (Supplementary Figures S3F–J). *G3BP2* found correlated with *LRPPRC*, *YTHDC2*, *YTHDF2*, *YTHDF3*, *KIAA1429*, and *WTAP* by Spearman correlation analysis (|R| > 0.4, *p* < 0.001) (Supplementary Figures S3K–P). In summary, for the three outcomes-related m6A regulators, we believe that *METTL14* has the strongest regulation on outcomes, including the regulation of lung function, and positively correlates with m6A writers and readers to promote RNA methylation, followed by *G3BP2*, which is also positively correlated to m6A writers and readers.

### 3.3 Two m6A patterns identified by prognosis-related m6A regulators

**FIGURE 4:**
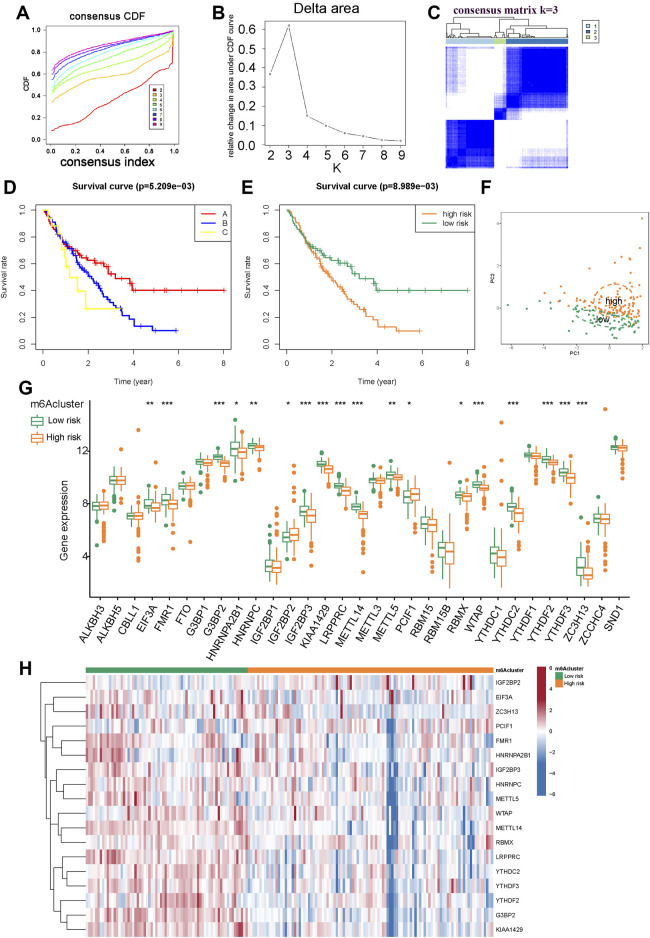
Consensus clustering of prognosis-related m6A modulators in IPF. **(A)** The CDF plot. **(B)** The relative change in area under the CDF curve. **(C)** Consensus matrixes of the two significant m6A regulators (*METTL14* and *G3BP2*) for k = 3. **(D)** Kaplan-Meier plot of overall survival in different m6A clusters. **(E)** Kaplan-Meier plot of overall survival in m6A low-risk (cluster A) and m6A high-risk (cluster B and C). **(F)** PCA for m6A low-risk and high-risk. **(G)** Histogram of 31 m6A regulators in m6A low-risk and m6A high-risk. **(H)** Expression heatmap of 18 differentially expressed m6A regulators in m6A low-risk and high-risk.

We applied the “consensus clustering method” to explore the impact of different m6A patterns on prognosis based on three m6A prognostic regulators. Compared with clusters 1 to 9, the growth rate in cluster two was flat in the CDF plot (Supplementary Figure S4A). Kaplan-Meier survival analysis of IPF patients with two different m6A clusters showed that the prognosis of clusters A and B were not significant (*p* = 0.123) (Supplementary Figure S4B). We believe that this m6A model does not correlate well with IPF outcomes. According to these conclusions, we selected *METTL14* and *G3BP2* with strong prognostic correlations as variables to distinguish m6A patterns. Compared with clusters 1 to 9, the growth rate in cluster three was flat in the CDF plot (Figure 4A). Figure 4B shows that the relative change in area under the CDF curve increased insignificantly for cluster 3. In the consistency matrix of cluster 3, the intra-group correlation was higher, and the inter-group correlation was low (Figure 4C and Supplementary Figures S4D–J). In summary, we identified three m6A patterns. Kaplan-Meier survival analysis of IPF patients showed significant differences in outcomes among the three clusters (*p* = 0.005) (Figure 4D). Clusters B (89 cases) and C (17 cases) had poor prognosis; therefore, we believe that cluster A (70 cases) is the m6A low-risk group (containing 70 cases); clusters B and C are the m6A high-risk group (containing 106 cases) for Kaplan-Meier survival analysis. The low-risk group had significantly better outcomes than the high-risk group (*p* = 0.008) (Figure 4E). The PCA scatter diagram was obtained for visualization, which distinguished between m6A low-risk and high-risk (Figure 4F). Boxplots and heatmaps of m6A-related genes were drawn based on different m6A risk groups to demonstrate the differential expression levels of 31 m6A regulators (Figures 4G,H); 18 m6A regulators were significantly differentially expressed.

### 3.4 Identification of DEGs and enrichment of molecular mechanisms and biological functions in different m6A risk groups

**FIGURE 5:**
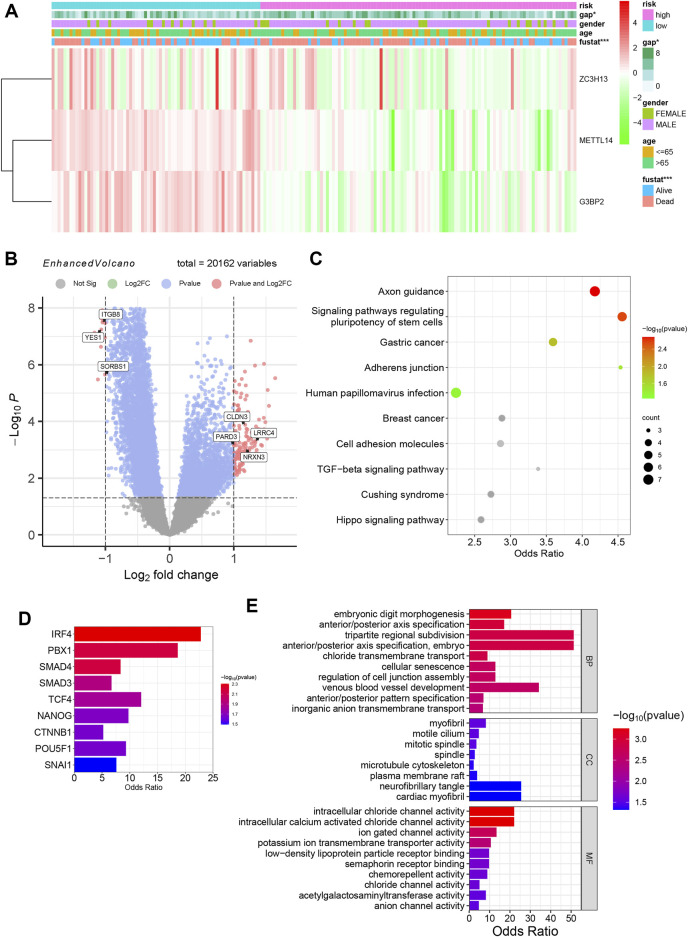
Biological characteristics of different m6A risk groups. **(A)** Heatmap of m6A risk groups with clinical information. (*p < 0.05; ***p < 0.001). **(B)** The enhanced volcano plot of DEGs between different m6A clusters. **(C)** KEGG pathway analysis of different m6A clusters. **(D)** Bar graph of transcription factor prediction using TRRUST. **(E)** GO annotation enrichment, including BP, CC, and MF analysis of various m6A clusters.

A clinical heatmap was drawn for the five prognostic m6A genes, gender, age (older than 65 years old), survival status, and GAP score data of patients in the m6A high- and low-risk groups (Figure 5A). The chi-square test was performed on the clinical information, and there was no significant difference in gender and age among patients between risk groups, while the survival status (*p* < 0.001) and GAP scores (*p* < 0.05) were significantly different. Using the “limma” package to analyze the DEGs of the m6A high-risk versus low-risk group, 195 DEGs were obtained (Supplementary Table S1), including 27 downregulated genes and 168 upregulated genes. Supplementary Figure S4K is a volcano plot of various m6A clusters, and Figure 5B is an enhanced volcano plot of various m6A risk groups, showing the primary DEGs and marking the genes related to cell adhesion. The KEGG pathway analysis enriched adherens junction and cell adhesion molecules (Figure 5C). *YES1*, *ITGB8*, *SORBS1*, *CLDN3*, *LRRC4*, *NRXN3*, and *PARD3* were DEGs related to adherens junction and cell adhesion molecules. Three downregulated genes and four upregulated genes in the high-risk group are shown in Figure 5B. Functions related to cell adhesion were observed in the GO annotation. Regulation of cell junction assembly was the affected BP (Figure 5E). These findings suggest that cell adhesion and junction functions might be regulated by m6A regulators, mediating IPF progression. Using TRRUST prediction, SMAD4 and SMAD3 were the main transcription factors of the TGF-β/SMAD pathway and are considered the essential pathway for IPF progression (Figure 5D). Myofibril and microtubule cytoskeleton were the affected CCs associated with fibroblasts.

### 3.5 Immune infiltration analysis of different m6A patterns

**FIGURE 6:**
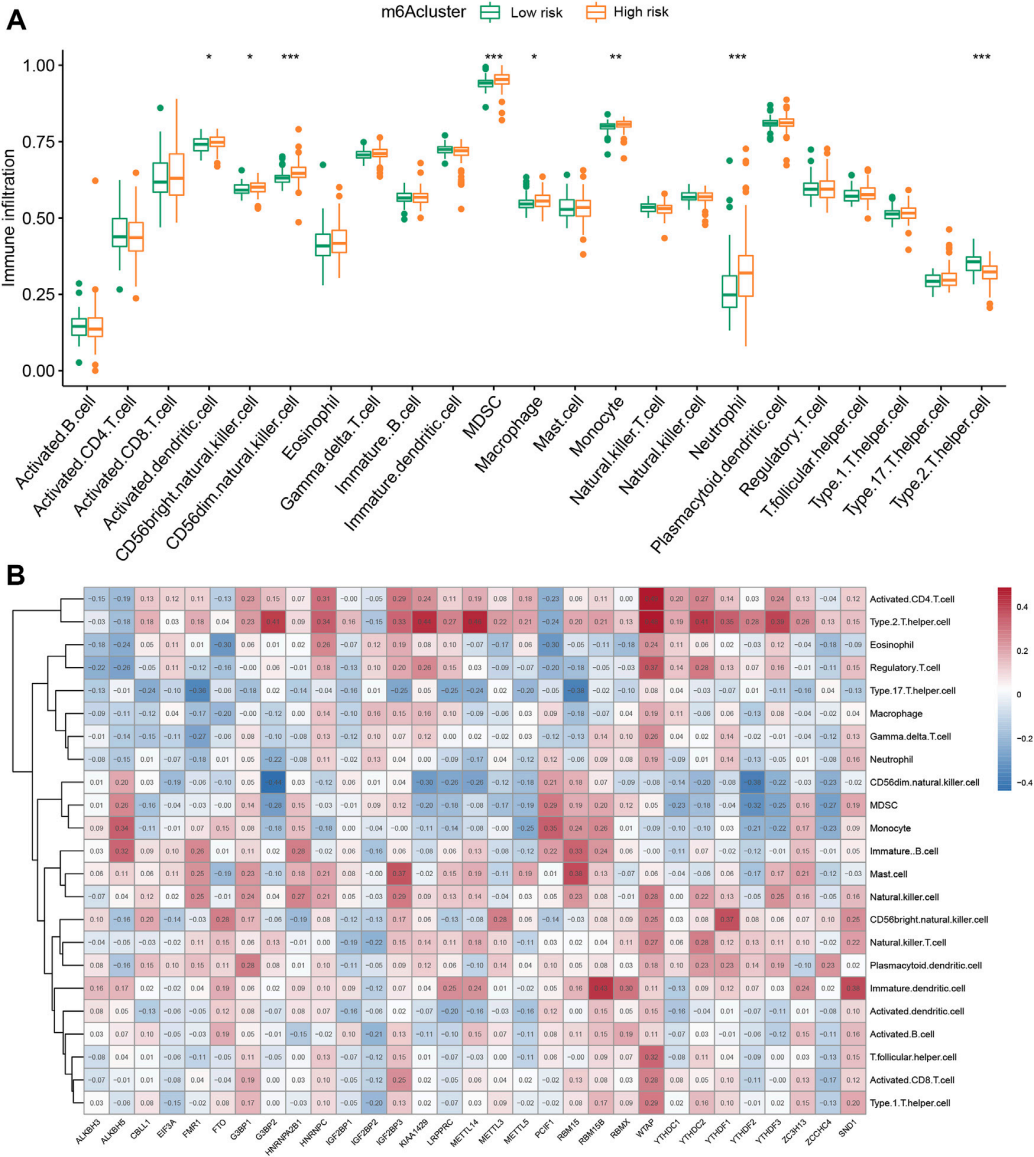
Single-sample gene set enrichment analysis. **(A)** Twenty-three immune cell infiltration differences among various m6A risks (*p < 0.05, **p < 0.01, ***p < 0.001). **(B)** Correlations between infiltrating immune cells and 31 m6A regulators.

To explore the influence of immune cells on IPF patients with different m6A types, we applied ssGSEA to visualize the abundance of immune cells in IPF samples. We found that neutrophils in the high-risk group were significantly upregulated compared with the low-risk group. Clinical study also showed that the upregulation of neutrophils signals poor IPF outcomes (Jegal, 2022). The high-risk group was markedly enriched in activated CD56dim natural killer cells, myeloid-derived suppressor cells, and type 2 T helper cells (Figure 6A). We applied Spearman correlation analysis to assess the relationships between m6A regulators and immune cell infiltration (Figure 6B). We evaluated the correlation between three m6A regulators regulating immune cells and found that *G3BP2* and *METTL14* were m6A regulators with negative correlations with neutrophil expression. We also explored differential immune cell infiltration between patients with high and low *G3BP2* or *METTL14* expression (Supplementary Figures S5A,B). *WTAP* is an m6A methyltransferase, and various immune cells positively correlate with *WTAP*. *WTAP* upregulates T cells, including activated CD4 T cell, regulatory T cell, T follicular helper cell, and type 2 T helper cell (Supplementary Figures S5C).

### 3.6 Potential drug targets for m6A clusters

**FIGURE 7:**
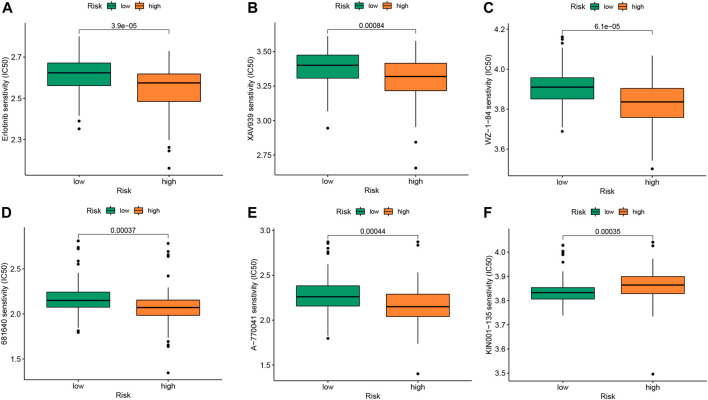
Drug sensitivity analysis of IPF patients in different m6A risk group. **(A)** Erlotinib **(B)** XAV939 **(C)** WZ-1-84 **(D)** 681640 **(E)** A-770041 **(F)** KIN001-135. The comparison of the drug sensitivity between m6A low- and high-risk groups. Lower IC_50_ indicates more sensitivity.

The “pRRophetic” package is a computational model that predicts chemotherapy responses based on tumor gene expression data (Altmann et al., 2010). IPF drugs are associated with the development of anti-tumor drugs such as nintedanib, an oral small molecule tyrosine kinase inhibitor initially developed for lung cancer and approved for the treatment of IPF. Pirfenidone has a sensitizing effect on the combination of paclitaxel and carboplatin used in clinical practice (Branco et al., 2022). We believe that the identification of related anti-tumor drugs has potential significance for IPF treatment. We obtained six drugs potentially associated with m6A based on a two-sided *p*-value <0.01. Drugs sensitive to the m6A high-risk group included erlotinib, XAV939, WZ-1-84, 681640, and A-770041 (Figures 7A–E). KIN001-135 was more sensitive to the m6A low-risk group (Figures 7F). The m6A low-risk group had better prognosis, while the m6A high-risk group was associated with more potentially sensitive drugs.

## 4 Discussion

We explored the significance of m6A regulators in IPF diagnosis and prognosis. First, we explored the models by which different m6A modification patterns are associated with IPF diagnosis and identified five diagnostic m6A regulators from a lung tissue-derived dataset, including *PCIF1*, *RBM15B*, *CBLL1*, *SND1*, and *FMR1*. The diagnostic accuracy was subsequently validated on BALF and PBMC-derived datasets. We believe that the five diagnostical m6A regulators have diagnostic significance, and it is possible to collect PBMCs from whole blood for diagnosis; nevertheless, more clinical samples are needed for verification.

We then explored the m6A regulators associated with IPF outcomes and identified *METTL14*, *G3BP2*, and *ZC3H13* as m6A-associated prognostic protective factors. The risk score calculated by the LASSO regression model and the GAP score were identified as independent m6A prognostic factors. Next, to predict the regulatory patterns of prognosis-related m6A, we performed consistent clustering using *METTL14* and *G3BP2* and identified three subgroups with significantly different prognosis, among which subgroups B and C had poor outcomes; therefore, we considered subgroup A as the m6A low-risk group, and subgroups B and C as the m6A high-risk groups.

Fibrogenesis aggravates pulmonary fibrosis, and TGF-β/SMAD is a critical pathway for fibrosis (Chanda et al., 2019; Kadota et al., 2021). SMAD3 and SMAD4 were predicted to be significant differential transcription factors across risk groups. Enhanced tight junction function can maintain the integrity and plasticity of alveolar epithelial cells (Varma et al., 2014). Tight junction and adherens junction proteins are upregulated in regenerated alveolar epithelial cells after pulmonary fibrosis, suggesting that enhanced cell adhesion function is beneficial to the prognosis of fibrosis (Lappi-Blanco et al., 2013). Tight junctions provide a physical barrier to epithelial cells and regulate the flow between cells (Zou et al., 2020). METTL14 is an m6A methyltransferase complex that stabilizes the structure and recognizes target RNA (Liu et al., 2018). We speculate that the upregulation of cell adhesion-related proteins in the low-risk group is related to the stabilization of cell adhesion-related RNA by METTL14.

**FIGURE 8:**
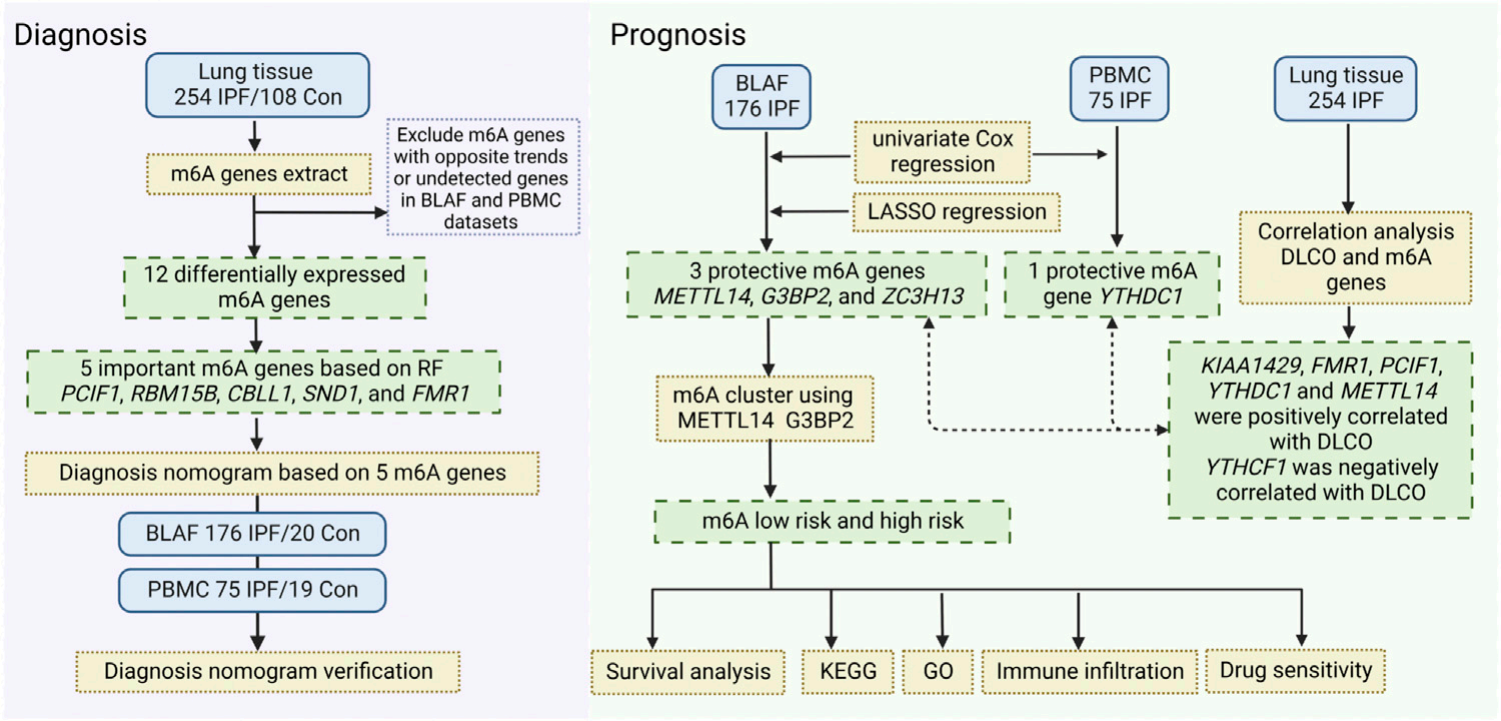
The flow chart of the contents of the project.

We then analyzed the expression of immune cells in different m6A risk groups and found that neutrophils were significantly upregulated in the high-risk group. Studies linked neutrophils to pulmonary fibrosis disease. Clinical studies showed that blood neutrophil levels positively correlated with IPF progression (Achaiah et al., 2021; Nathan et al., 2021), and the ratio of neutrophils in the blood of IPF patients was inversely proportional to forced vital capacity and forced expiratory volume in one second (Nathan et al., 2021; D'Alessandro et al., 2022). A cohort study of 156 IPF patients identified a high proportion of neutrophils in BALF as an independent predictor of early death (Jegal, 2022). Therefore, the upregulation of neutrophils can be considered a reliable prognostic risk factor in IPF patients, associated with shortened overall survival (Chen et al., 2022). A flowchart outlines the contents of the project (Figure 8).

## 5 Conclusion

We assessed m6A modification patterns in 505 IPF patients and 147 healthy volunteers based on m6A modulators and identified five diagnostic m6A modulators. Based on clinical data, we identified prognosis-related m6A regulators in IPF. *METTL14* and *G3BP2* were the critical m6A regulators for prognosis that divided IPF patients into m6A types with different risks and revealed the biological mechanisms behind different m6A modification patterns. m6A modification may play a role in stabilizing tight junction-associated mRNA. There was upregulation of neutrophil expression in the m6A high-risk group, which indicated poor outcomes. Finally, we tested the drug sensitivity of various risk types. These findings provide a basis for the study of IPF-related m6A regulation.

## Data Availability

Publicly available datasets were analyzed in this study. This data can be found here: https://www.ncbi.nlm.nih.gov/geo/query/acc.cgi?acc=GSE70866; https://www.ncbi.nlm.nih.gov/geo/query/acc.cgi?acc=GSE47460; https://www.ncbi.nlm.nih.gov/geo/query/acc.cgi?acc=GSE28221.

## Abbreviations

IPF, idiopathic pulmonary fibrosis; m6A, N6-methyladenosine; GEO, Gene Expression Omnibus; BLAF, bronchoalveolar lavage fluid; PBMC, peripheral blood mononuclear cell; PCA, principal component analysis; RF, random forest; SVM, support vector machine; DCA, decision curve analysis; ROC, receiver operating characteristic; AUC, area under the curve; CI, confidence interval; LASSO, least absolute shrinkage and selection operator; GAP, global alignment and proportion; DLCO, diffusing capacity of the lungs for carbon monoxide; CDF, cumulative distribution function; DEGs, differentially expressed genes; GO, Gene Ontology; KEGG, Kyoto Encyclopedia of Genes and Genomes; BP, biological processes; CC, cellular components; MF, molecular function; ssGSEA, single-sample gene set enrichment analysis.
