# Supplementary material for: Genes related to N6-methyladenosine in the diagnosis and prognosis of idiopathic pulmonary fibrosis
Source: Front Genet. 2023 Jan 4;13:1102422. doi: 10.3389/fgene.2022.1102422 (PMC9846232; doi:10.3389/fgene.2022.1102422)
Supplement: Supplementary file 2 [file DataSheet1.docx]

Supplementary Material


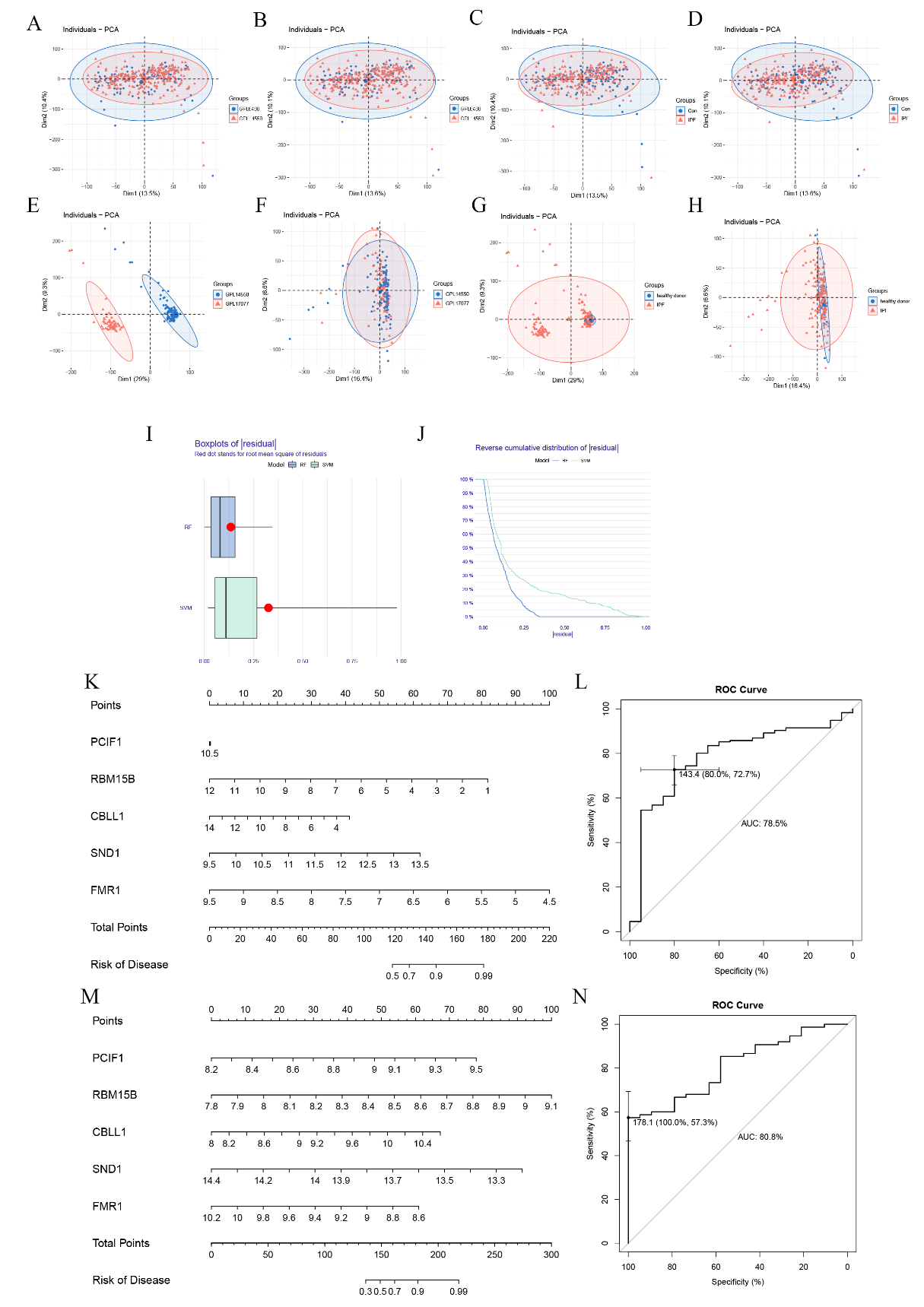


**Supplementary Figure 1.** (A-D) Eliminating the batch effect between different sequencing platforms in GSE47460 series. (E-H) Eliminating the batch effect between different sequencing platforms in GSE70866 series. (I) Boxplots of residual was plotted to show the residual distribution of RF and SVM model. (J) Reverse cumulative distribution of |residual| of the RF and SVM model. (K-L) Nomogram and ROC curves of predicted prevalence according to gene score of GSE70866 series. (M-N) Nomogram and ROC curves of predicted prevalence according to gene score of GSE28221 series.


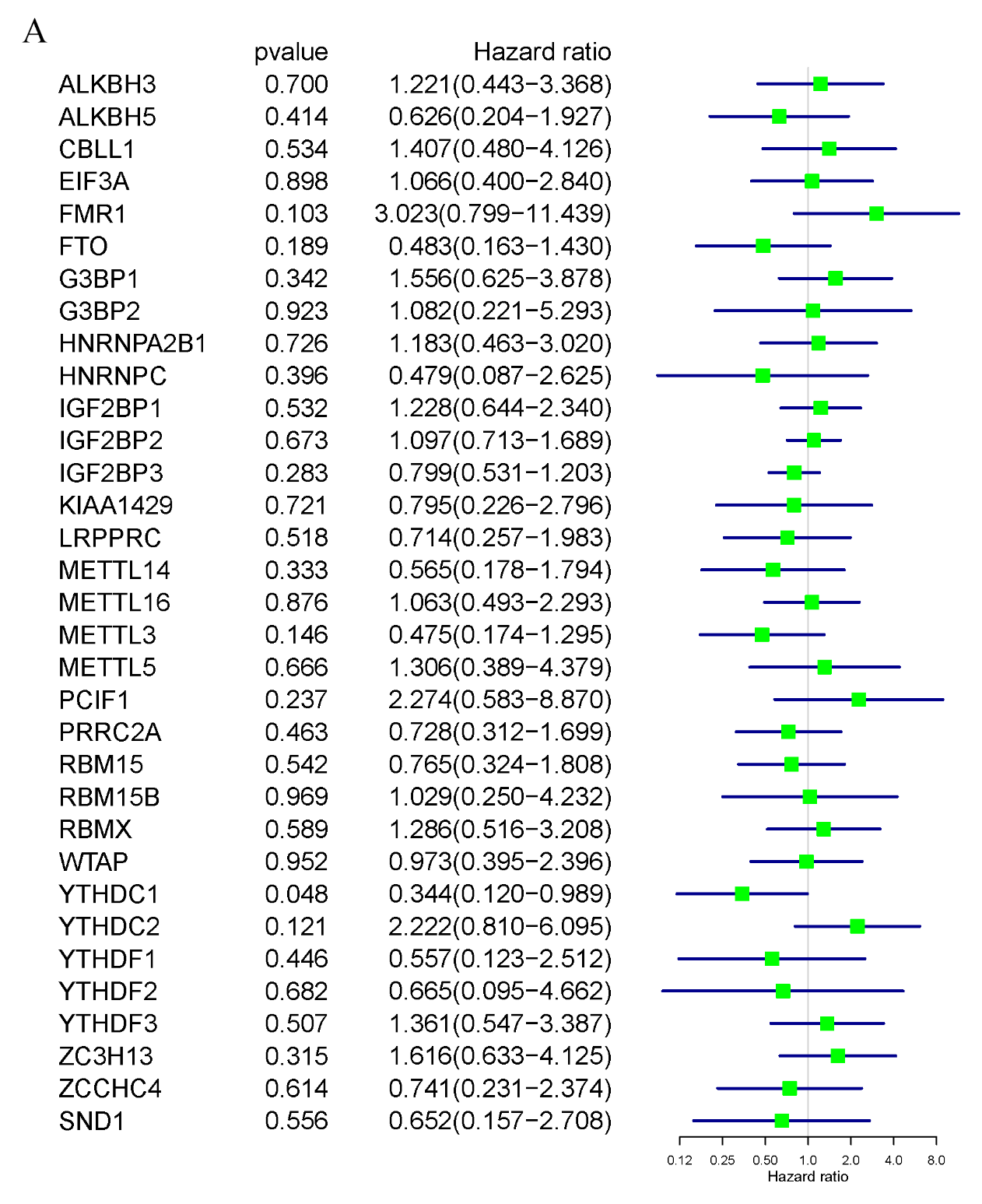


**Supplementary Figure 2.** (A) Forest plot of 1 m6A regulators with P < 0.05 by univariate Cox regression. (GSE28221 series)


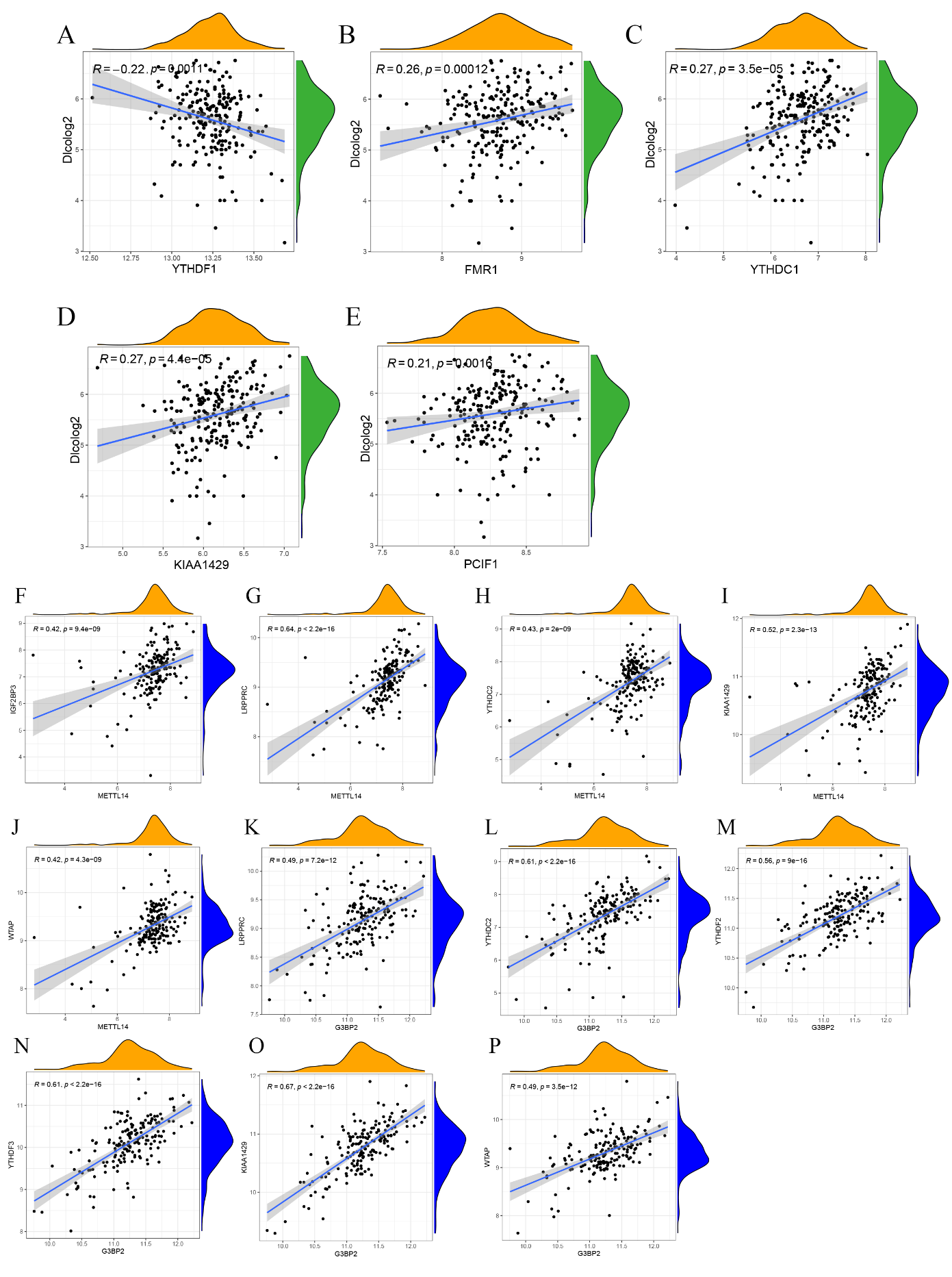


**Supplementary Figure 3.** (A-E) Correlation between m6A regulators and DLCO. (|R| > 0.2 and p < 0.001). (F-J) Correlation analysis of *METTL14* with m6A regulators. (K-P) Correlation analysis of *ZC3H13* with m6A regulators.


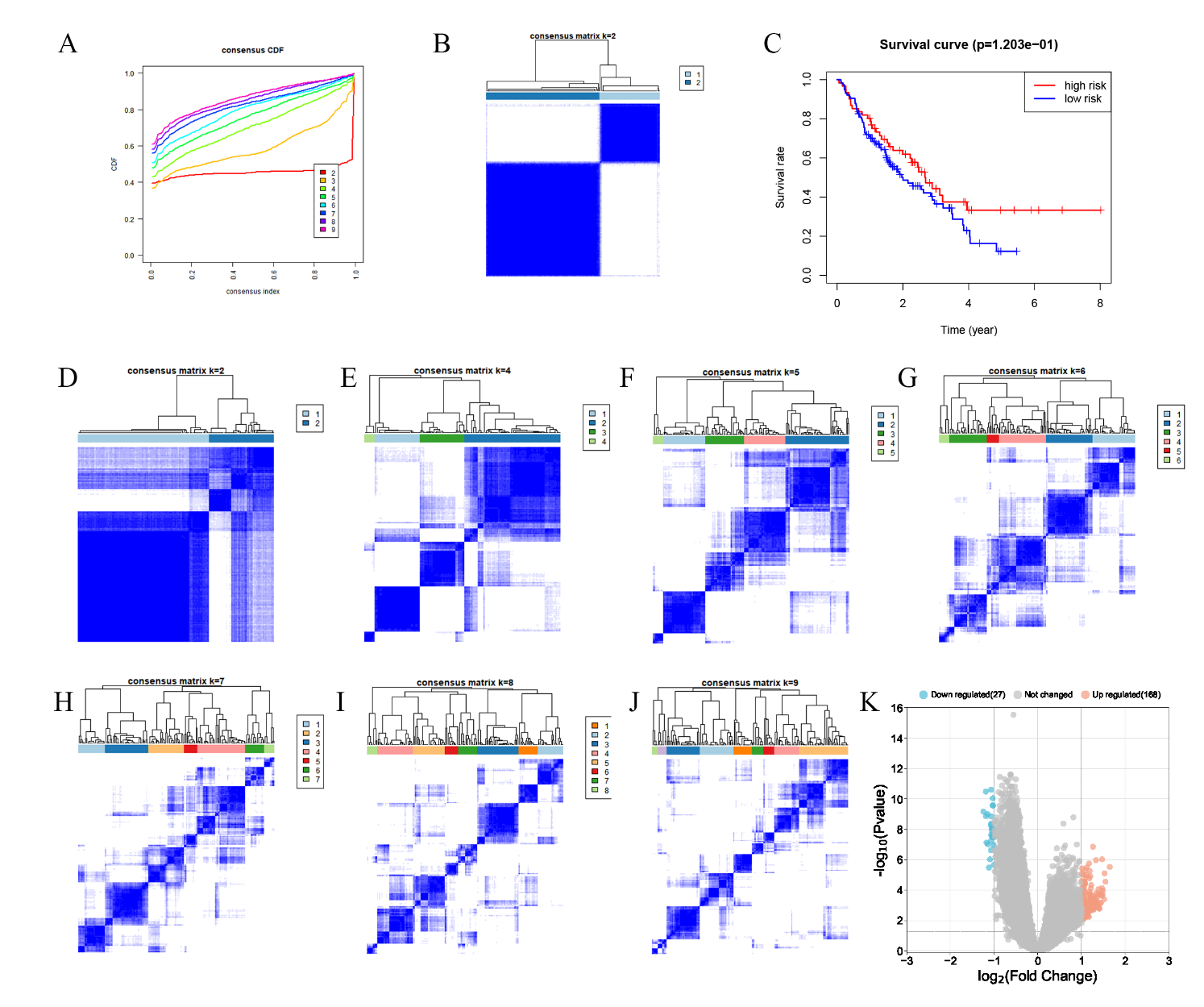


**Supplementary Figure 4.** (A) The CDF plot demonstrates that among 2–9. (B) Consensus matrices of the 3 significant m6A regulators (*METTL14,* *G3BP2*, and *ZC3H13*) for k = 2. (C) Kaplan–Meier plot of overall survival in different m6A clusters. (D-G) Consensus matrices of the 3 significant m6A regulators (*METTL14* and *G3BP2*) for k = 2, 4-9 (K) The volcano plot of DEGs between different m6A risk group.


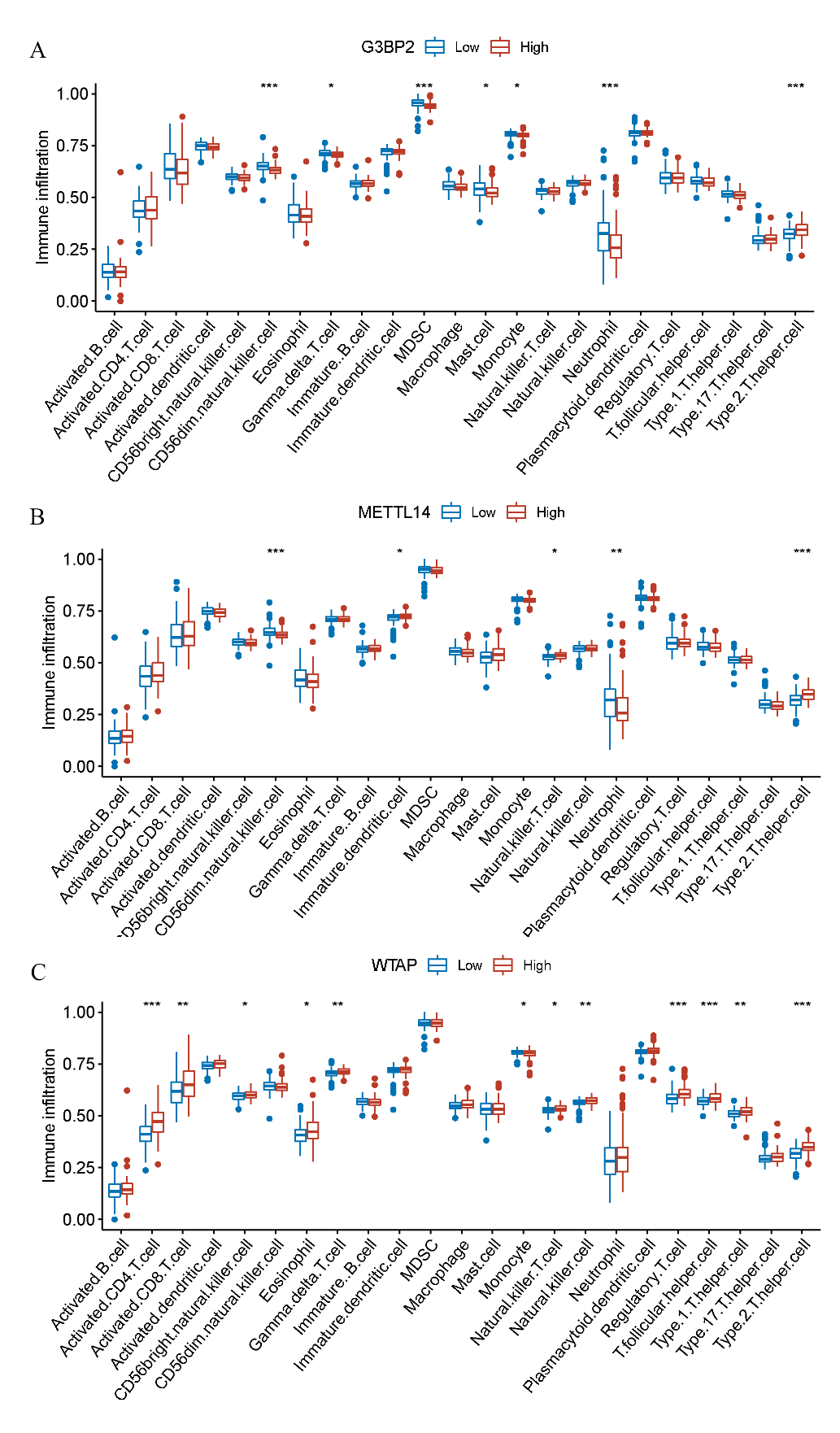


**Supplementary Figure 5.** (A) Differential immune cell infiltration between low *G3BP2* expression group and high *G3BP2* expression group. (B) Differential immune cell infiltration between low *METTL14* expression group and high *METTL14* expression group. (C) Differential immune cell infiltration between low *WTAP* expression group and high *WTAP* expression group. *p < 0.05, **p < 0.01, and ***p < 0.001.

**Supplementary Table 1.** 195 DEGs between high-risk and low-risk m6A risk group.
